# Obstetrical mode of delivery and behavioural outcomes in childhood and adolescence: findings from the Millennium Cohort Study

**DOI:** 10.1007/s00127-022-02233-x

**Published:** 2022-01-15

**Authors:** Gillian M. Maher, Ali S. Khashan, Fergus P. McCarthy

**Affiliations:** 1 INFANT Research Centre, Cork, Ireland; 2 School of Public Health, University College Cork, Cork, Ireland; 3 Department of Obstetrics and Gynaecology, University College Cork, Cork, Ireland

**Keywords:** Mode of delivery, Caesarean section, Behavioural outcomes, Epidemiology, Millennium Cohort Study

## Abstract

**Purpose:**

To examine the association between mode of delivery (in particular caesarean section) and behavioural outcomes in offspring at six time-points between age 3 and 17 years.

**Methods:**

Similar to previous work examining the association between mode of delivery and behavioural outcomes in offspring at age 7, we used maternal-reported data from the Millennium Cohort Study. Data on mode of delivery were collected when children were 9 months and categorised as spontaneous vaginal delivery, assisted vaginal delivery, induced vaginal delivery, emergency caesarean section, planned caesarean section and caesarean section after induction of labor. Data on behavioural outcomes were collected at ages 3, 5, 7, 11, 14 and 17 years using the Strengths and Difficulties Questionnaire (SDQ). Crude and adjusted logistic regression examined mode of delivery–behavioural difficulties relationship, using validated SDQ cut-off points (total SDQ ≥ 17, emotional ≥ 5, conduct ≥ 4, hyperactivity ≥ 7, peer problems ≥ 4 and prosocial behaviour ≤ 4). Multilevel models with linear splines examined the association between mode of delivery and repeated measures of SDQ.

**Results:**

There were 18,213 singleton mother–child pairs included at baseline, 13,600 at age 3; 13,831 at age 5; 12,687 at age 7; 11,055 at age 11; 10,745 at age 14 and 8839 at age 17. Adjusted logistic regression suggested few associations between mode of delivery and behavioural outcomes at ages 3, 5, 11, 14 and 17 years using validated SDQ cut-off points. After correction for multiple testing, only the protective association between planned caesarean section-Conduct difficulties at age 5 years (OR 0.63, 95% CI 0.46, 0.85) and positive association between caesarean section after induction-Emotional difficulties at age 11 years (OR 1.57, 95% CI 1.19, 2.07) remained statistically significant. Multilevel modelling suggested mean SDQ scores were similar in each mode of delivery group at each time point.

**Conclusions:**

Results of this study indicate that mode of delivery is unlikely to have a major impact on behavioural outcomes.

**Supplementary Information:**

The online version contains supplementary material available at 10.1007/s00127-022-02233-x.

## Introduction

Caesarean section rate is increasing worldwide. Between 2000 and 2015, birth by caesarean section has increased from ~12 to ~21% globally but with considerable variations between countries driven by factors such as income level of country, increases in the proportion of births occurring in health institutions and women’s education [1]. In Western Europe rate of caesarean section increased from almost 20 to 27% between 2000 and 2015, while the observed increase in Eastern Europe and central Asia was 12–27% in the same time period [1].

While caesarean section is vital to reduce maternal and neonatal morbidity and mortality when indicated [1, 2], less is known of its benefits in the absence of obstetric complications, and conversely has been linked to various adverse outcomes such as asthma [3], obesity [4], type 1 diabetes [5] and type 2 diabetes [4]. Furthermore, caesarean section has been linked to behavioural disorders in the offspring which may negatively impact child and family life and lead to impairments in personal, social, academic, or occupational functioning [6]. Previous studies suggest a small increase in the likelihood of attention deficit hyperactivity disorder [7], autism spectrum disorder [8], and adverse behavioural outcomes [9] in children delivered via caesarean section, with the concept of a microbiota–gut–brain axis often cited as the mechanism linking caesarean section to neurodevelopment [10]. For example, caesarean section has been shown to lead to differences in the intestinal microbiota of babies, which in turn may influence gut–brain communication, brain function and behaviour potentially through neural, endocrine and immune pathways [10–12]. However, evidence is conflicting, possibly due to different measures of behavioural outcomes (such as self-reported and clinical diagnoses), varying degrees of adjustment for potential confounding factors and different ranges in follow-up [13–16], In addition to this, few studies take account of repeated measures of behaviour over time despite evidence that behavioural issues are not always stable, and children can sometimes transition in or out of the cut-off range for behavioural outcomes [17]. Using a British cohort, we have previously examined the association between mode of delivery and behavioural outcomes at age 7 only. This study showed an effect among those born via induced vaginal delivery, though results attenuated after controlling for several important confounders [18]. Further research on this cohort, allowing for changes in behaviour over time, is warranted to inform pregnant women, their partners and clinicians of the potential effects of caesarean section on behavioural outcomes in the offspring.

Using data from the Millennium Cohort study, the aim of this study was to examine the association between mode of delivery (in particular caesarean section) and behavioural outcomes using the Strengths and Difficulties Questionnaire (SDQ) at ages 3, 5, 7, 11, 14 and 17 years using validated cut-off points, while also taking account of repeated measures of SDQ to allow for change in SDQ score over time.

## Methods

### Study population

The Millennium Cohort Study (MCS) is a nationally representative longitudinal study of children born between 2000 and 2002, and living in 398 areas in England, Scotland, Wales and Northern Ireland. There have been seven sweeps of the MCS participants to date. Sweep one (MCS1) of data collection took place when children were around 9 months old, and follow-up have currently been conducted at ages 3 years (MCS2), 5 years (MCS3), 7 years (MCS4), 11 years (MCS5), 14 years (MCS6) and 17 years (MCS7) [19, 20]. Ethical approval was obtained from an NHS Research Ethics Committee (MREC) and has, therefore, been performed in accordance with the ethical standards laid down in the 1964 Declaration of Helsinki and its later amendments. Informed consent was obtained from parents prior to inclusion in the study, as well as from the children themselves as they grow up [19].

### Sampling frame

The MCS used stratified cluster sampling by UK country (England, Wales, Scotland and Northern Ireland), and electoral ward. Eligible children were identified using government child benefit records as these records have close to universal coverage (with the exception of families whose residency status is temporary or uncertain, for example, members of foreign armed forces or asylum seekers). Intentional oversampling of certain subgroups of the population took place to ensure that typically hard to reach populations were adequately represented. These included children living in disadvantaged areas, children of ethnic minority backgrounds and children growing up in the smaller nations of the UK. Cohort members remained eligible for inclusion if they remained living in the United Kingdom (UK) at the time of sampling. The total population at baseline (MCS1) was 18,552 families (18,827 children) [19, 21]. This reduced to 15,590 families at MCS2, 15,246 at MCS3, 13,857 at MCS4, 13,287 at MCS5, 11,726 at MCS6 and 10,625 at MCS7 [19, 20].

### Exposure

*Mode of delivery:* data on mode of delivery were obtained when children were around 9 months old (MCS1) through a face-to-face computer assisted personal interview. The majority of main respondents (99.7%) were the infant’s biological mother [18]. Mode of delivery was categorised into “spontaneous vaginal delivery,” “assisted vaginal delivery,” “induced vaginal delivery,” “emergency caesarean section,” “planned caesarean section” and “caesarean section after induction”. The variable was defined this way to assess the effect of any intervention on labor as compared to no intervention (i.e. spontaneous, non-assisted vaginal delivery) [18]. Those that reported “normal delivery” or “water birth,” and responded “no” to the question “was the labor induced or attempted to be induced” were considered “spontaneous vaginal delivery”. Those that reported “assisted with forceps,” “assisted vacuum extraction,” “assisted breach” or “other assisted delivery” were considered “assisted vaginal delivery”. Those that reported “normal delivery” or “water birth,” but responded “yes” to the question “was the labor induced or attempted to be induced” were considered “induced vaginal delivery.” Respondents who reported an “emergency caesarean section” or “planned caesarean section” were recorded as such. However, those that reported “emergency caesarean section,” or “planned caesarean section” but responded “yes” to the question “was the labor induced or attempted to be induced” were considered “caesarean section after induction”. Attempted and successful inductions were grouped together, as the women were exposed to the same intervention. Answers that were recorded as “other” or “irrelevant response” were considered missing.

### Outcome

#### Strengths and Difficulties Questionnaire (SDQ)

The SDQ consists of a 25-item questionnaire with 5 subscales: emotional, conduct, hyperactivity, peer problems and prosocial behaviours. It was developed as a screening tool to assess emotional and behavioural problems in children and young people between 2 and 16 years old [22], while it is now also used as a behavioural screening questionnaire for people in the age range 2–18 years[23]. A slightly modified version is available for 3-year-old children, whereby the item on reflectiveness is softened, and two items on antisocial behaviour are replaced by items on oppositionality [23]. For the current study, data were collected using parent-administered SDQ when children were aged 3 years (MCS2), 5 years (MCS3), 7 years (MCS4), 11 years (MCS5) 14 years (MCS6) and 17 years (MSC7).

The main respondent (which was usually the mother) answered “not true”, “somewhat true”, and “certainly true” to a series of questions, with ‘somewhat true’ always scored as 1, and the scoring of ‘not true’ and ‘certainly true’ varying with the item (full scoring procedures are available online: https://www.sdqscore.org). Scores for each domain range from 0 to 10, with lower scores indicating more positive outcomes, with the exception of prosocial behaviour which is a reversed score (i.e. higher scores indicate more positive outcomes). Similar to other childhood behavioural outcome studies conducted on Millennium Cohort participants [22, 24], SDQ cut-off points for behavioural difficulties were defined as follows: total SDQ ≥ 17 (total score was generated by summing scores from all the scales except the prosocial scale, and classified as missing if one of the four component scores was missing), emotional ≥ 5, conduct ≥ 4, hyperactivity ≥ 7, peer problems ≥ 4 and prosocial behaviour ≤ 4. These cut-offs correspond to approximately 90th percentile of the distribution of the SDQ scores according to the British non-age or gender-specific norms [22].

### Confounding variables

We included only covariates in our model, which we believed to be associated with the exposure and outcome, and have excluded any variables that might be potential mediators of the association. Therefore, we controlled for the following potential confounders, all of which were measured at baseline (MCS1): maternal age, maternal education, maternal smoking status, maternal alcohol consumption during pregnancy, pre-pregnancy body mass index (BMI), household income, small for gestational age (SGA), infant sex, parity, hypertensive disorders of pregnancy (including raised blood pressure, eclampsia/preeclampsia or toxaemia) and maternal depression/serious anxiety. Additionally, we stratified results by level of deprivation and excluded preterm births in separate analyses. We also stratified results by presence or absence of maternal depression/serious anxiety to further explore its impact on results. A description of potential confounders is as follows:

*Maternal age:* measured in years and referred to mother’s age at time of birth of cohort member. *Maternal education:* highest academic qualification was categorised as higher degree, first degree, diploma in higher education, A/AS/S levels, O level/GCSE grades A-C, GCSE grades D-G, other academic qualifications and none of these qualifications. Higher degree, first degree and diploma in higher education were recategorised as diploma or above. A/AS/S levels were recategorised as A level. O level/GCSE grades A-C and GCSE grades D-G were recategorised as O level. Other academic qualifications were recategorised as other/unknown and none of these qualifications were recategorised as less than O level. *Maternal smoking status:* mothers were asked the number of cigarettes they smoked per day before pregnancy and the number smoked per day if they changed the amount smoked during pregnancy. This information was recategorised as non-smoker, quit during pregnancy and smoked during pregnancy. *Maternal alcohol consumption during pregnancy:* mothers were asked about their frequency of alcohol consumption during pregnancy. This information was recategorised as a dichotomous variable (maternal alcohol consumption during pregnancy: yes/no). *Pre-pregnancy BMI:* maternal height and weight just before becoming pregnancy were self-reported; these were used to calculate maternal BMI and was recategorised as underweight, normal weight, overweight, obese and unknown. *Household income:* categorised according to the Organisation for Economic Co-operation and Development (OECD) income weighted quintiles. *Small for gestational age *(*SGA*)*:* defined as birthweight < 10th percentile for gestational age and sex of child and based on maternal-reporting of child’s birthweight, gestational age and sex. *Infant sex:* categorised as male/female. *Parity:* derived from number of cohort member’s siblings in the household and recategorised as primiparous/multiparous. *Hypertensive disorders of pregnancy:* mothers were asked: “Did you have any illnesses or other problems during your pregnancy that required medical attention or treatment?” If the answer to this question was yes, she was instructed to choose all that apply from a list of illnesses. The list included “Raised blood pressure, eclampsia/preeclampsia or toxaemia”. If she ticked this box, then a diagnosis of hypertensive disorders of pregnancy was assumed. *Maternal depression/serious anxiety:* mothers were asked whether a doctor ever told them that they suffer from depression or serious anxiety. This information was categorised as yes/no. *Level of deprivation:* deprivation decile scores were calculated from home postcodes using the 2004 overall indices of multiple deprivation (IMD) [25]. A binary variable was created to represent children living in areas of highest deprivation (deciles 1–5) and lowest deprivation (deciles 6–10). *Preterm birth:* mothers were asked about their child’s gestation age in days. This was converted to weeks and recategorised as <37 weeks’ gestation and ≥ 37 weeks’ gestation.

### Statistical analysis

Data were analysed using Stata/MP 14.2. Survey commands were used, and estimates were weighted to account for the stratified cluster sample design and analyses covering the whole of the UK. Crude and adjusted logistic regression analysis estimated odds ratios (OR) and 95% confidence intervals (CI) for mode of delivery and behavioural difficulties using SDQ cut-off points at ages 3, 5 and 7, 11, 14 and 17 years. Bonferroni correction was used when a result was statistically significant to adjust for multiple tests. We divided the original α-value of 0.05 by 6 (i.e. the number of analyses on the outcome at each age group) producing a more conservative significance level of 0.008.

*Repeated measures analysis:* As the SDQ was measured at six time points (ages 3, 5 and 7, 11, 14 and 17 years), multilevel modelling with linear splines (placing ‘knot points’ at age 5 and 7, 11, 14 and 17 years) was used to take account of repeated measures over time. The multilevel approach can estimate the SDQ trajectory for all participants regardless of the number and timing of their measurements. This approach also takes non-linearity in the trajectory into account. This is an important function of multilevel modelling as associations are not always linear, although it is an assumption of the standard linear regression [26]. In addition, multilevel models address the issue of correlations between measurements from the same individual over time as they take non-independence of repeated measures on the same individual into account [27, 28]. We modelled trajectories for mode of delivery and SDQ score on a continuous scale, with random effects at two levels: measurement occasion and individual. The starting point was centred at age 3 (when SDQ was first measured) and all models adjusted for potential confounding factors, as outlined above.

*Sensitivity analysis:* As area-level deprivation may influence mode of delivery [29], we stratified mode of delivery-total SDQ cut-off results by level of deprivation. In addition, we repeated the main analysis examining mode of delivery-total SDQ cut-off while excluding preterm births (gestational age < 37 weeks’), as those born preterm may be less likely to be delivered via planned caesarean section. As maternal anxiety and depression may increase the likelihood of caesarean section [30], we stratified results by presence or absence of maternal depression/serious anxiety. To examine the potential for selection bias as a result of loss to follow-up, we repeated the main adjusted analyses examining mode of delivery and total SDQ cut-off, among participants selected based on having complete exposure, covariate and outcome data at all time points. We examined the association between mother and child characteristics (including maternal age, maternal education, maternal smoking status, maternal alcohol consumption during pregnancy, pre-pregnancy BMI, household income, SGA, infant sex, parity, hypertensive disorders of pregnancy, maternal depression/serious anxiety and total SDQ cut-off at age 3 years) and loss to follow-up at age 17 years. We repeated the main analysis examining mode of delivery-total SDQ cut-off and included the attrition weight for age 17 to account for non-random loss to follow-up. Finally, we recategorised mode of delivery into a binary variable: (1) any vaginal delivery (reference group) and (2) any caesarean section, and examined the effect on total SDQ cut-off. All sensitivity analyses are included in a supplementary online file.

## Results

A total of 18,213 singleton mother–child pairs with data on mode of delivery were included in the current study (i.e. 492 children were excluded due to multiple births). Mother and child characteristics are outlined in Table 1. Of the study cohort, spontaneous vaginal delivery was reported in 48.68% (*n* = 8866) participants, assisted vaginal delivery in 9.53% (*n* = 1735), induced vaginal delivery in 20.52% (*n* = 3739), emergency caesarean section in 6.88% (*n* = 1253), planned caesarean section in 7.91% (*n* = 1440), and caesarean section after induction in 6.48% (*n* = 1180) participants.

**Table 1:** Mother and child characteristics related to mode of delivery and childhood behavioural outcomes among Millennium Cohort Study participants

Characteristic	Spontaneous VD	Assisted VD	Induced VD	Emergency CS	Planned CS	CS after induction
Total population, *N* (%)	8866 (48.68)	1735 (9.53)	3739 (20.52)	1253 (6.88)	1440 (7.91)	1180 (6.48)
Maternal age, years, mean (SD)	27.92 (5.89)	28.06 (5.81)	27.69 (6.07)	29.30 (5.88)	30.75 (5.34)	29.14 (5.95)
Maternal education completed, *n* (%)						
Less than O level	1853 (20.90)	198 (11.41)	841 (22.49)	208 (16.60)	247 (17.15)	191 (16.19)
O level	3887 (43.84)	751 (43.29)	1718 (45.95)	524 (41.82)	636 (44.17)	513 (43.47)
A level	811 (9.15)	187 (10.78)	344 (9.20)	126 (10.06)	114 (7.92)	111 (9.41)
Diploma or above	2008 (22.65)	559 (32.22)	721 (19.28)	358 (28.57)	406 (28.19)	326 (27.63)
Other/unknown	307 (3.46)	40 (2.31)	115 (3.08)	37 (2.95)	37 (2.57)	39 (3.31)
Maternal smoking status, *n* (%)						
Non-smoker	5691 (64.21)	1137 (65.61)	2266 (60.64)	827 (66.00)	984 (68.33)	785 (66.53)
Quit during pregnancy	1055 (11.90)	277 (15.98)	479 (12.82)	158 (12.61)	176 (12.22)	170 (14.41)
Smoked during pregnancy	2117 (23.89)	319 (18.41)	992 (26.55)	268 (21.39)	280 (19.44)	225 (19.07)
Maternal alcohol consumption during pregnancy, *n* (%)						
Yes	2612 (29.47)	513 (29.57)	1026 (27.46)	333 (26.58)	432 (30.00)	321 (27.20)
Maternal pre-pregnancy BMI, *n* (%)						
Underweight	529 (5.97)	84 (4.84)	231 (6.18)	73 (5.83)	47 (3.26)	42 (3.56)
Normal weight	5497 (62.00)	1135 (65.42)	2127 (56.89)	677 (54.03)	746 (51.81)	621 (52.63)
Overweight	1483 (16.73)	310 (17.87)	715 (19.12)	257 (20.51)	315 (21.88)	261 (22.12)
Obese	539 (6.08)	103 (5.94)	336 (8.99)	129 (10.30)	178 (12.36)	153 (12.97)
Unknown	818 (9.23)	103 (5.94)	330 (8.83)	117 (9.34)	154 (10.69)	103 (8.73)
Household income, *n* (%)						
Lowest quintile	2391 (27.05)	311 (17.95)	1084 (29.16)	262 (21.04)	281 (19.54)	228 (19.34)
Second quintile	2118 (23.96)	283 (16.33)	901 (24.23)	251 (20.16)	287 (19.96)	242 (20.53)
Third quintile	1627 (18.41)	344 (19.85)	712 (19.15)	238 (19.12)	296 (20.58)	226 (19.17)
Fourth quintile	1466 (16.59)	377 (21.75)	553 (14.87)	240 (19.28)	289 (20.10)	239 (20.27)
Highest quintile	1236 (13.99)	418 (24.12)	468 (12.59)	254 (20.40)	285 (19.82)	244 (20.70)
Small for gestational age, *n* (%)	790 (8.91)	136 (7.84)	410 (10.97)	170 (13.57)	93 (6.46)	139 (11.78)
Infant sex, *n* (%)						
Male	4480 (50.53)	956 (55.10)	1870 (50.01)	721 (57.54)	705 (48.96)	636 (53.90)
Parity (first born child), *n* (%)	3047 (34.37)	1356 (78.16)	1423 (38.06)	693 (55.31)	350 (24.31)	781 (66.19)
Hypertensive disorders of pregnancy, *n* (%)	326 (3.68)	160 (9.22)	346 (9.25)	150 (11.97)	123 (8.54)	192 (16.27)
Maternal depression/serious anxiety, *n* (%)	2061 (23.25)	400 (23.05)	1013 (27.09)	302 (24.10)	398 (27.64)	299 (25.34)
Total SDQ, mean (SD)						
Age 3 years (*N* = 13,600)	9.63 (5.30)	9.35 (4.84)	9.86 (5.51)	9.33 (5.14)	9.12 (5.17)	9.55 (5.36)
Age 5 years (*N* = 13,831)	7.37 (4.98)	7.14 (4.83)	7.45 (5.09)	7.53 (5.13)	6.88 (4.72)	7.13 (5.12)
Age 7 years (*N* = 12,687)	7.44 (5.43)	7.20 (5.13)	7.73 (5.64)	7.54 (5.43)	7.14 (5.12)	7.41 (5.54)
Age 11 years (*N* = 11,055)	7.37 (5.72)	7.15 (5.26)	7.76 (5.74)	7.81 (5.93)	7.14 (5.43)	7.69 (5.85)
Age 14 years (*N* = 10,745)	8.17 (6.01)	7.56 (5.62)	8.52 (6.04)	8.13 (5.88)	7.74 (5.85)	8.11 (5.81)
Age 17 years (*N* = 8839)	7.90 (6.19)	7.73 (6.12)	8.23 (6.37)	7.82 (6.14)	7.55 (6.17)	7.68 (5.91)

*VD* vaginal delivery, *CS* caesarean section, *BMI* body mass index, *SDQ* Strengths and Difficulties Questionnaire, *SD* standard deviation

### Mode of delivery and SDQ (ages 3, 5, 7, 11, 14 and 17 years)

*Logistic regression:* Adjusted results in Table 2 suggested that planned caesarean section was associated with a 33% reduction in odds of having Prosocial Behaviour difficulties at age 3 years compared to spontaneous vaginal delivery (OR 0.67, 95% CI 0.48, 0.94). No other significant associations were observed at age 3. At age 5, emergency caesarean section was associated with a 49% increase in odds of having behavioural difficulties based on total SDQ score (OR 1.49, 95% CI 1.04, 2.11) and a 39% increase in odds of having peer problem difficulties (OR 1.39, 95% CI 1.03, 1.87). Conversely, planned caesarean section was associated with a 37% reduction in odds of having Conduct difficulties (OR 0.63, 95% CI 0.46, 0.85) at age 5 years. No significant associations were observed at age 7 years.

**Table 2:** Association between mode of delivery and domains of the Strengths and Difficulties Questionnaire at ages 3, 5, 7, 11, 14 and 17 years among Millennium Cohort Study participants

	No. in each exposure group with SDQ ≥ 17	Total SDQ (cut-off ≥ 17) OR (95% CI)^*a*^	Emotional OR (95% CI)^*a*^	Conduct OR (95% CI)^*a*^	Hyperactivity OR (95% CI)^*a*^	Peer problems OR (95% CI)^*a*^	Prosocial behaviour OR (95% CI)^*a*^
Age 3 years							
Spontaneous VD (*N* = 6571)	710	Ref	Ref	Ref	Ref	Ref	Ref
Assisted VD (*N* = 1348)	107	0.91 (0.68, 1.20)	1.13 (0.82, 1.55)	1.01 (0.85, 1.18)	0.93 (0.75, 1.15)	0.98 (0.78, 1.23)	0.98 (0.72, 1.34)
Induced VD (*N* = 2741)	350	1.02 (0.86, 1.21)	1.04 (0.84, 1.29)	0.99 (0.88, 1.10)	0.93 (0.80, 1.08)	0.92 (0.79, 1.09)	0.88 (0.70, 1.10)
Emergency CS (*N* = 942)	83	0.80 (0.58, 1.09)	1.21 (0.87, 1.70)	0.85 (0.70, 1.03)	0.94 (0.73, 1.20)	0.95 (0.74, 1.23)	1.19 (0.85, 1.66)
Planned CS (*N* = 1112)	102	0.89 (0.67, 1.17)	0.99 (0.70, 1.39)	0.94 (0.79, 1.11)	1.01 (0.81, 1.27)	0.85 (0.66, 1.09)	0.67 (0.48, 0.94)^*^
CS after induction (*N* = 886)	99	1.29 (0.95, 1.74)	1.25 (0.88, 1.77)	0.97 (0.80, 1.18)	1.00 (0.78, 1.29)	0.71 (0.54, 0.93)	0.69 (0.47, 1.03)
Age 5 years							
Spontaneous VD (*N* = 6714)	372	Ref	Ref	Ref	Ref	Ref	Ref
Assisted VD (*N* = 1370)	66	1.08 (0.77, 1.52)	1.25 (0.92, 1.71)	0.84 (0.64, 1.10)	0.98 (0.77, 1.26)	1.03 (0.77, 1.38)	0.74 (0.41, 1.34)
Induced VD (*N* = 2810)	187	1.11 (0.89, 1.38)	0.92 (0.72, 1.17)	0.88 (0.74, 1.04)	0.95 (0.80, 1.13)	1.09 (0.89, 1.34)	1.08 (0.75, 1.55)
Emergency CS (*N* = 937)	66	1.49 (1.04, 2.11)^*^	1.08 (0.74, 1.55)	0.86 (0.63, 1.17)	1.30 (0.99, 1.70)	1.39 (1.03, 1.87)^*^	1.41 (0.81, 2.47)
Planned CS (*N* = 1102)	44	0.73 (0.48, 1.11)	0.93 (0.64, 1.35)	0.63 (0.46, 0.85)^*^	0.91 (0.69, 1.21)	0.93 (0.68, 1.28)	0.87 (0.51, 1.47)
CS after induction (*N* = 898)	51	1.10 (0.75, 1.63)	1.12 (0.77, 1.62)	0.86 (0.63, 1.17)	0.80 (0.59, 1.08)	1.15 (0.83, 1.59)	1.00 (0.54, 1.87)
Age 7 years							
Spontaneous VD (*N* = 6161)	450	Ref	Ref	Ref	Ref	Ref	Ref
Assisted VD (*N* = 1249)	82	0.81 (0.58, 1.12)	1.04 (0.77, 1.40)	0.95 (0.72, 1.26)	0.84 (0.66, 1.07)	1.20 (0.92, 1.57)	0.60 (0.33, 1.09)
Induced VD (*N* = 2572)	226	1.13 (0.92, 1.39)	1.00 (0.81, 1.23)	1.10 (0.92, 1.32)	1.02 (0.86, 1.20)	1.18 (0.98, 1.43)	0.76 (0.50, 1.15)
Emergency CS (*N* = 877)	65	0.88 (0.61, 1.27)	1.01 (0.71, 1.42)	0.79 (0.57, 1.10)	1.22 (0.94, 1.59)	1.09 (0.79, 1.50)	0.74 (0.38, 1.44)
Planned CS (*N* = 1016)	66	1.01 (0.72, 1.42)	0.99 (0.71, 1.38)	1.02 (0.77, 1.35)	0.94 (0.72, 1.21)	0.87 (0.64, 1.18)	0.56 (0.28, 1.12)
CS after induction (*N* = 812)	55	0.88 (0.61, 1.28)	0.97 (0.69, 1.37)	0.91 (0.66, 1.25)	0.87 (0.65, 1.17)	0.99 (0.72, 1.37)	0.99 (0.53, 1.87)
Age 11 years							
Spontaneous VD (*N* = 5391)	431	Ref	Ref	Ref	Ref	Ref	Ref
Assisted VD (*N* = 1099)	76	0.94 (0.68, 1.29)	1.09 (0.84, 1.42)	0.67 (0.48, 0.94)^*^	0.99 (0.74, 1.30)	1.07 (0.82, 1.39)	0.78 (0.43, 1.43)
Induced VD (*N* = 2193)	191	0.98 (0.79, 1.22)	1.00 (0.83, 1.21)	0.97 (0.79, 1.18)	1.13 (0.94, 1.37)	1.03 (0.85, 1.24)	1.20 (0.79, 1.83)
Emergency CS (*N* = 754)	73	1.20 (0.86, 1.67)	1.13 (0.85, 1.49)	1.12 (0.81, 1.53)	1.39 (1.05, 1.84)^*^	1.32 (1.01, 1.75)^*^	0.66 (0.29, 1.46)
Planned CS (*N* = 892)	55	0.77 (0.54, 1.11)	1.16 (0.89, 1.52)	0.86 (0.63, 1.18)	0.93 (0.69, 1.25)	0.90 (0.66, 1.21)	0.79 (0.36, 1.75)
CS after induction (*N* = 726)	60	1.10 (0.76, 1.59)	1.57 (1.19, 2.07)^*^	0.85 (0.60, 1.21)	0.90 (0.64, 1.26)	1.44 (1.08, 1.90)^*^	0.93 (0.47, 1.85)
Age 14 years							
Spontaneous VD (*N* = 5310)	526	Ref	Ref	Ref	Ref	Ref	Ref
Assisted VD (*N* = 1036)	90	0.93 (0.69, 1.25)	0.91 (0.70, 1.18)	1.02 (0.76, 1.37)	0.81 (0.59, 1.11)	1.00 (0.79, 1.27)	0.89 (0.61, 1.29)
Induced VD (*N* = 2114)	229	1.07 (0.87, 1.31)	1.02 (0.85, 1.22)	0.99 (0.81, 1.21)	1.00 (0.81, 1.23)	1.02 (0.86, 1.20)	0.72 (0.52, 0.98)
Emergency CS (*N* = 747)	68	0.98 (0.70, 1.38)	1.18 (0.89, 1.56)	0.79 (0.56, 1.11)	1.10 (0.78, 1.53)	1.09 (0.85, 1.42)	1.03 (0.67, 1.58)
Planned CS (*N* = 851)	75	1.03 (0.75, 1.42)	0.92 (0.69, 1.21)	1.09 (0.80, 1.48)	0.93 (0.67, 1.29)	0.85 (0.66, 1.11)	0.85 (0.52, 1.36)
CS after induction (*N* = 687)	67	1.03 (0.73, 1.43)	1.37 (1.04, 1.80)^*^	0.87 (0.62, 1.22)	1.13 (0.80, 1.58)	1.04 (0.79, 1.35)	1.04 (0.66, 1.64)
Age 17 years							
Spontaneous VD (*N* = 4367)	442	Ref	Ref	Ref	Ref	Ref	Ref
Assisted VD (*N* = 894)	81	1.06 (0.78, 1.45)	1.14 (0.88, 1.47)	0.96 (0.70, 1.31)	1.42 (1.01, 1.99)^*^	0.87 (0.68, 1.11)	1.23 (0.81, 1.86)
Induced VD (*N* = 1671)	196	1.19 (0.96, 1.48)	0.93 (0.77, 1.13)	1.17 (0.93, 1.47)	1.09 (0.83, 1.43)	0.90 (0.75, 1.08)	1.06 (0.73, 1.52)
Emergency CS (*N* = 638)	58	1.08 (0.75, 1.56)	1.15 (0.87, 1.53)	0.78 (0.53, 1.16)	0.95 (0.61, 1.47)	1.10 (0.84, 1.43)	0.51 (0.26, 1.00)
Planned CS (*N* = 692)	60	1.26 (0.89, 1.78)	1.27 (0.97, 1.66)	1.22 (0.86, 1.73)	1.00 (0.65, 1.53)	1.04 (0.80, 1.36)	0.84 (0.46, 1.53)
CS after induction (*N* = 577)	49	1.04 (0.71, 1.50)	1.20 (0.90, 1.62)	0.81 (0.54, 1.21)	0.94 (0.58, 1.52)	0.80 (0.60, 1.08)	1.04 (0.60, 1.80)

*SDQ* Strengths and Difficulties Questionnaire, *OR* odds ratio, *95% CI* 95% confidence interval, *VD* vaginal delivery, *CS* caesarean section

^*^Statistically significant *p* < 0.05

^a^Adjusted for maternal age, maternal education, maternal smoking status, maternal alcohol consumption during pregnancy, pre-pregnancy body mass index, household income, small for gestational age, infant sex, parity, hypertensive disorders of pregnancy and maternal depression/serious anxiety

At age 11 years, caesarean section after induction was associated with an increased odds of emotional difficulties (OR 1.57, 95% CI 1.19, 2.07) and peer problem difficulties (OR 1.44, 95% CI 1.08, 1.90). Similarly, emergency caesarean section was associated with an increased odds of Hyperactivity difficulties (OR 1.39, 95% CI 1.05, 1.84) and Peer Problem difficulties (OR 1.32, 95% CI 1.01, 1.75). However, assisted vaginal delivery was associated with a reduction in odds of having Conduct difficulties at age 11 years (OR 0.67, 95% CI 0.48, 0.94).

At age 14 years, caesarean section after induction was associated with an increased odds of Emotional difficulties (OR 1.37, 95% CI 1.04, 1.80). Finally, assisted vaginal delivery was associated with an increase in odds of Hyperactivity difficulties at age 17 years (OR 1.42, 95% CI 1.01, 1.99). However, after Bonferroni correction, only the estimate for planned caesarean section-Conduct difficulties at age 5 years (OR 0.63, 95% CI 0.46, 0.85), and caesarean section after induction-Emotional difficulties at age 11 years (OR 1.57, 95% CI 1.19, 2.07) remained statistically significant (i.e. result was still significant at (0.05/6) *p* < 0.008). Unadjusted and confounder adjusted results were not materially different (Table S1 in the supplementary online file).

**Fig. 1:**
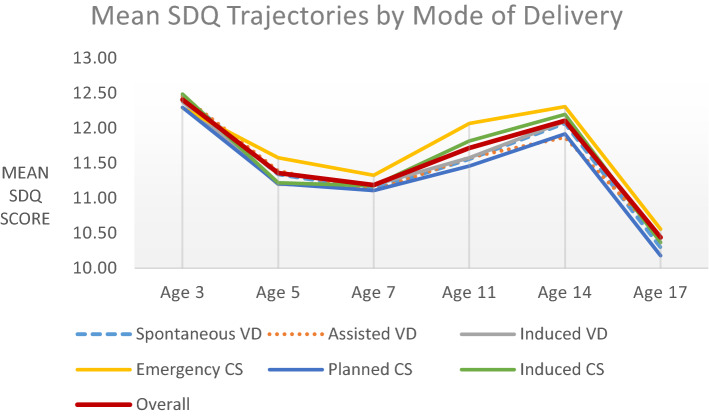
Predicted trajectory of mean SDQ scores at ages 3, 5, 7, 11, 14 and 17 years overall and by mode of delivery (adjusted model)

*Repeated measures analysis:* Adjusted mean SDQ trajectories overall and by mode of delivery from age 3 to 17 years are shown in Fig. 1. From age 3 to 5 years and 5 to 7 years, mean SDQ scores decreased in all of the exposure groups. From age 7 to 11 years and 11 to 14 years, mean SDQ scores increased in all exposure groups. Finally, from age 14 to 17 years, mean SDQ scores decreased again in all exposure groups. Mean SDQ score in each mode of delivery group were not significantly different at any time point (Table 3).

**Table 3:** Repeated measures analysis examining the association between mode of delivery and total Strengths and Difficulties Questionnaire score at ages 3, 5, 7, 11, 14 and 17 years among Millennium Cohort Study participants

	Mean trajectory (95% CI) spontaneous VD	Mean trajectory (95% CI) assisted VD	Mean trajectory (95% CI) induced VD	Mean trajectory (95% CI) emergency CS	Mean trajectory (95% CI) planned CS	Mean trajectory (95% CI) CS after induction
Model 1^a^						
Age 3 SDQ	9.78 (9.67, 9.90)	9.42 (9.17, 9.68)	10.00 (9.82, 10.18)	9.48 (9.18, 9.79)	9.18 (8.90, 9.46)	9.63 (9.32, 9.95)
Change SDQ age 5	−2.28 (−2.17, −2.39)	−2.21 (−1.96, −2.45)	−2.38 (−2.21, 2.55)	−1.82 (−1.53, −2.12)	−2.15 (−1.88, −2.42)	−2.37 (−2.07, −2.67)
Change SDQ age 7	0.09 (−0.01, 0.20)	0.12 (−0.11, 0.36)	0.31 (0.15, 0.48)	0.14 (−0.14, 0.43)	0.16 (−0.09, 0.43)	0.34 (0.04, 0.63)
Change SDQ age 11	0.15 (0.03, 0.27)	0.13 (−0.13, 0.40)	0.15 (−0.03, 0.35)	0.48 (0.15, 0.80)	0.18 (−0.11, 0.48)	0.40 (0.06, 0.74)
Change SDQ age 14	0.59 (0.46, 0.72)	0.25 (−0.03, 0.53)	0.62 (0.42, 0.82)	0.22 (−0.11, 0.55)	0.53 (0.22, 0.84)	0.37 (0.03, 0.72)
Change SDQ age 17	−0.09 (−0.24, 0.05)	0.14 (−0.18, 0.46)	−0.01 (−0.24, 0.22)	−0.16 (−0.54, 0.22)	−0.03 (−0.40, 0.33)	−0.24 (−0.65, 0.15)
Age 17 SDQ	8.24 (8.07, 8.44)	7.85 (7.46, 8.27)	8.69 (8.42, 9.00)	8.34 (7.86, 8.83)	7.87 (7.42, 8.33)	8.13 (7.63, 8.64)
Model 2^b^						
Age 3 SDQ	12.45 (10.35, 14.56)	12.46 (10.35, 14.57)	12.38 (10.27, 14.48)	12.29 (10.18, 14.40)	12.30 (10.19, 14.42)	12.49 (10.38, 14.60)
Change SDQ Age 5	−1.11 (−3.41, 1.76)	−1.07 (−3.37, 1.22)	−1.18 (−3.47, 1.11)	−0.71 (−3.01, 1.58)	−1.09 (−3.39, 1.21)	−1.27 (−3.57, 1.03)
Change SDQ Age 7	−0.21 (−2.42, 1.98)	−0.29 (−2.50, 1.90)	−0.01 (−2.21, 2.19)	−0.25 (−2.45, 1.95)	−0.10 (−2.32, 2.10)	−0.04 (−2.25, 2.16)
Change SDQ Age 11	0.43 (−2.16, 3.03)	0.48 (−2.12, 3.08)	0.39 (−2.20, 2.99)	0.74 (−1.86, 3.35)	0.35 (−2.25, 2.96)	0.64 (−1.96, 3.24)
Change SDQ Age 14	0.51 (−2.15, 3.18)	0.29 (−2.38, 2.97)	0.52 (−2.14, 3.19)	0.24 (−2.43, 2.92)	0.46 (−2.21, 3.14)	0.38 (−2.29, 3.06)
Change SDQ Age 17	−1.77 (−4.65, 1.11)	−1.41 (−4.30, 1.47)	−1.67 (−4.55, 1.20)	−1.75 (−4.65, 1.13)	−1.74 (−4.64, 1.14)	−1.83 (−4.73, 1.06)
Age 17 SDQ	10.30 (6.85, 13.73)	10.46 (7.00, 13.89)	10.43 (7.00, 13.87)	10.56 (7.11, 14.00)	10.18 (6.71, 13.62)	10.37 (6.91, 13.81)

*SDQ* Strengths and Difficulties Questionnaire, *95% CI* 95% confidence interval, *VD* vaginal delivery, *CS* caesarean section

^a^Crude analysis

^b^Adjusted for maternal age, maternal education, maternal smoking status, maternal alcohol consumption during pregnancy, pre-pregnancy body mass index, household income, small for gestational age, infant sex, parity, hypertensive disorders of pregnancy and maternal depression/serious anxiety

*Sensitivity analysis:* Similar to our main findings, adjusted estimates suggested a significant association between emergency caesarean section and total SDQ cut-off at age 5 years among those in the lowest deprivation deciles only (OR: 2.18, 95% CI: 1.08, 4.40). At age 7, induced vaginal delivery was associated with an increased odds of behavioural difficulties based on total SDQ score among those in the highest deprivation deciles only (OR 1.33, 95% CI 1.01, 1.74). However, after Bonferroni correction, these associations were no longer significant (Table S2 in the supplementary online file). Results of the sensitivity analysis excluding preterm births were not materially different to our main findings (Table S3 in the supplementary online file).

Among those whose mothers did not have depression/serious anxiety, adjusted estimates suggested a significant association between caesarean section after induction and total SDQ cut-off at age 3 years (OR 1.45, 95% CI 1.02, 2.06). Among those whose mothers had depression/serious anxiety, an association between emergency caesarean section and total SDQ cut-off was observed (OR 2.04, 95% CI 1.18, 3.50) at age 5 years, while an association between induced vaginal delivery (OR 1.77, 95% CI 1.22, 2.57) and planned caesarean section (OR 2.06, 95% CI 1.15, 3.68) was observed at age 17 years. However, after Bonferroni correction, only the association for induced vaginal delivery at age 17 remained significant (Table S4 in the supplementary online file). Finally, adjusted associations of mode of delivery and total SDQ cut-off, among participants selected based on having complete exposure, covariate and outcome data were not significantly different to our main findings (Table S5 in the supplementary online file). Loss to follow-up by age 17 years was significantly associated with younger maternal age, lower levels of maternal education, maternal smoking, reporting no alcohol consumption during pregnancy, maternal underweight, lower household income, SGA, male infant sex, maternal depression/serious anxiety and total SDQ cut-off at age 3 years (Table S6 in the supplementary online file). Including the attrition weight for age 17 did not materially change results (Table S7 in the supplementary online file). When compared to any vaginal delivery, caesarean section (emergency, planned or caesarean section after induction combined) was not significantly associated with total SDQ cut-off at any time point (Table S8 in the supplementary online file).

## Discussion

We have previously examined the association between mode of delivery and behavioural outcomes at age 7 only [18]. The current study extended beyond this by investigating the association between mode of delivery (in particular caesarean section) and behavioural outcomes using the SDQ at ages 3, 5, 7, 11, 14 and 17 years using cut-off points, while also taking account of repeated measures of SDQ on a continuous scale in order to allow for change in SDQ score over time. We have yielded two principal findings.

First, only few associations between mode of delivery and behavioural outcomes were observed using SDQ cut-off points. However, after correction for multiple testing only two associations remained statistically significant; planned caesarean section was associated with a 37% reduction in odds of having Conduct difficulties at age 5 years and caesarean section after induction was associated with an 57% increased odds of Emotional difficulties at age 11 years. Nevertheless, it is also not possible to rule out the role of chance here, especially considering the lack of consistency across age groups and in direction of effect.

Second, the repeated measures analysis suggested that mean SDQ scores were similar in each mode of delivery group at each time point (ages 3, 5, 7, 11, 14 and 17 years), with the following trajectories observed: mean SDQ scores decreased in all of the exposure groups from age 3 to 5 years and 5 to 7 years. Mean SDQ scores increased in all exposure groups from age 7 to 11 years and 11 to 14 years. Finally, mean SDQ scores decreased again in all exposure groups from age 14 to 17 years.

### Comparison with other studies

Comparable with our findings, no significant association was observed between caesarean section and internalizing (emotional and peer problems) and externalizing (conduct and hyperactivity) domains of the SDQ in a Czech Republic cohort in children of preschool age [31]. However, the authors did not distinguish between planned and emergency caesarean section due to a small sample size (*N* = 256) [31]. Another study found that children born by caesarean delivery on maternal request are less likely to have externalizing behavior problems, a finding similar to our study [32]. Al Khalaf et al. [33] found no significant association between mode of delivery and total SDQ score at age 3 years in an Irish cohort. However, unlike our findings an association between mode of delivery and the hyperactivity domain of the SDQ in children born by emergency caesarean section and instrumental vaginal delivery was observed.

Conversely, previous research conducted on a Chinese cohort has indicated an association between elective caesarean section without medical indication and total SDQ score and emotional difficulties at preschool age (3–5 years) [34]. Results from a separate Chinese cohort consisting of 8900 children from 35 preschools in four cities in East China suggested that elective and emergency caesarean section were associated with total SDQ score, while elective caesarean section only was associated with prosocial difficulties [9]. This is in contrast to our study that found planned caesarean section to be protective against prosocial behavioural difficulties, albeit our result was no longer significant when multiple testing was taken into account.

Despite the SDQ being a valid tool to assess emotional and behavioural problems in children between 2 and 17 years old [22], there is a lack of overall evidence investigating the relationship between mode of delivery and SDQ in older age groups. This is a shortcoming that should be considered in future research as lower demands on younger children to self-regulate behaviour, may result in parents being less likely to view and rate their child’s behavior as unusual or problematic at a younger age [35, 36]. Furthermore, as previous evidence suggests that children can sometimes transition in or out of the cut-off range for behavioural issues throughout childhood [17], future studies should consider methods to deal with repeated measures when examining behavioural difficulties over time. Finally, SDQ predictive algorithms have been shown to identify young people who have an increased likelihood of developing other neurodevelopmental outcomes by estimating an ‘unlikely’, ‘possible’ and ‘probable’ rating [37]. As the SDQ cut-offs used in the current study represent a ‘probable’ rating, it may be worthwhile to consider a mode of delivery–SDQ relationship using ‘possible’ ratings to identify those who may have a greater likelihood of other mental health outcomes in preadolescence.

## Strengths and limitations

This study has several limitations that should be considered. First, data on mode of delivery and potential confounders were self-reported and are subject to recall bias. However, maternal reporting of mode of delivery in the MCS has previously been compared to hospital records, and was found to be highly reliable, with an agreement of 94–98% [38]. Furthermore, agreement between maternal reported and antenatal records on lifestyle factors such as smoking pre-pregnancy and smoking during pregnancy has been shown to be good to very good 4–9 years post-delivery[39]. It is also worth noting that data on potential confounders was collected 9 months post-delivery in the current study and is, therefore, likely to be more accurate than data collected 4–9 years after pregnancy. Second, our outcome data measured using the SDQ relied on the subjective evaluation of the child’s parents, though the scale has been widely used and found to be valid and reliable screening tool to assess behavioural difficulties in children and young people [40]. Third, residual or unmeasured confounding including confounding due to shared genetics cannot be ruled out. Furthermore, confounding by indication for mode of delivery may have influenced findings as we do not know why an emergency or planned caesarean section was performed. Fourth, loss to follow-up may have introduced selection bias. For example, our sample size included 18,213 singleton mother–child pairs in MCS1, which reduced to 10,331 at MCS7 (of which 8839 had data on SDQ and mode of delivery). As participation in follow-up research can be influenced by several factors, and children with behavioural disorders are more likely to be lost to follow-up, this may have biased our results towards the null [41]. However, with the repeated measures approach, it was possible to estimate the SDQ trajectory for all participants regardless of the number and timing of their measurements, thus increasing power and efficiency [27]. Also, results from analyses with and without selection on complete exposure, covariate and outcome data were not significantly different, indicating a low likelihood of selection driven by missing data.

Several strengths should also be noted. First, we used data from a nationally representative contemporary cohort of children born in the UK between 2000 and 2002. Second, to our knowledge, this is the first study to examine the association between mode of delivery and behavioural outcomes at six time points, from early childhood to late teens. Furthermore, multilevel modelling with linear splines was used to take account of repeated measures of the SDQ over time, taking non-linearity in the trajectory into account and addressing the issue of correlations between measurements from the same individual over time [27, 28]. Third, data regarding behavioural outcomes were collected prospectively; therefore, recall bias was less likely to influence results. Finally, we controlled for a wide range of potential confounding factors, including maternal age, maternal education, maternal smoking status, maternal alcohol consumption during pregnancy, pre-pregnancy BMI, household income, SGA, infant sex, parity, hypertensive disorders of pregnancy and maternal depression/serious anxiety.

## Conclusion

We did not find strong evidence of associations between mode of delivery and behavioural outcomes overall. Few associations were observed at ages 3, 5, 11, 14 and 17 years; however, it is likely that many of these occurred as a result of chance. Our results can reassure future parents and clinicians that caesarean section is unlikely to have an effect on behavioural outcomes. However, caesarean section is not without its risks for mother and baby and should involve serious consideration in the absence of medical indications.

## Supplementary Information

Below is the link to the electronic supplementary material.

Supplementary file1 (DOCX 81 kb)

## Data Availability

Access to data and supporting documentation can be requested from the UK Data Service: https://beta.ukdataservice.ac.uk/datacatalogue/studies/study?id=4683

## Code availability

Data were analysed using Stata/MP 14.2 commands. Queries re commands can be directed to the corresponding author.
