# Supplementary material for: Obstetrical mode of delivery and behavioural outcomes in childhood and adolescence: findings from the Millennium Cohort Study
Source: Soc Psychiatry Psychiatr Epidemiol. 2022 Jan 15;57(8):1697–709. doi: 10.1007/s00127-022-02233-x (PMC9288955; doi:10.1007/s00127-022-02233-x)
Supplement: Supplementary file 1 — Supplementary file1 (DOCX 81 kb) [file 127_2022_2233_MOESM1_ESM.docx]

**Supplementary Online File**

**Article:** Obstetrical Mode of Delivery and Behavioural Outcomes in Childhood and Adolescence: Findings from the Millennium Cohort Study

**Journal:** Social Psychiatry and Psychiatric Epidemiology

**Authors:** Gillian M. Maher^1,2^ PhD, Ali S. Khashan^1,2^ PhD, Fergus P. McCarthy^1,3^ PhD

**Affiliations:** ^1^INFANT Research Centre, Cork, Ireland; ^2^School of Public Health, Western Gateway Building, University College Cork, Cork, Ireland; ^3^Department of Obstetrics and Gynaecology, University College Cork, Cork, Ireland

**Corresponding Author:** Dr Gillian M. Maher, Email: [gillian.maher@ucc.ie](mailto:gillian.maher@ucc.ie)

**Table S1:** Crude Association between Mode of Delivery and Domains of the Strengths and Difficulties Questionnaire at ages 3, 5, 7, 11, 14 and 17 years among Millennium Cohort Study Participants

**Table S2:** Association between Mode of Delivery and total Strengths and Difficulties Questionnaire cut-off at ages 3, 5, 7, 11, 14 and 17 years among Millennium Cohort Study Participants by Level of Deprivation

**Table S3:** Association between Mode of Delivery and total Strengths and Difficulties Questionnaire cut-off at ages 3, 5, 7, 11, 14 and 17 years among Millennium Cohort Study Participants (excluding preterm births)

**Table S4:** Association between Mode of Delivery and total Strengths and Difficulties Questionnaire cut-off at ages 3, 5, 7, 11, 14 and 17 years among Millennium Cohort Study Participants by Presence or Absence of Maternal Depression/Serious Anxiety

**Table S5:** Association between Mode of Delivery and total Strengths and Difficulties Questionnaire cut-off at ages 3, 5, 7, 11, 14 and 17 years among Millennium Cohort Study Participants (among participants selected based on having complete exposure, covariate and outcome data)

**Table S6:** Association between mother and child characteristics and loss to follow-up at age 17 years among Millennium Cohort Study Participants

**Table S7:** Association between Mode of Delivery and total Strengths and Difficulties Questionnaire cut-off at ages 3, 5, 7, 11, 14 and 17 years among Millennium Cohort Study Participants (including attrition weight for age 17)

**Table S8:** Association between any Caesarean Section and total Strengths and Difficulties Questionnaire cut-off at ages 3, 5, 7, 11, 14 and 17 years among Millennium Cohort Study Participants

| **Table S1: Crude Association between Mode of Delivery and Domains of the Strengths and Difficulties Questionnaire at ages 3, 5, 7, 11, 14 and 17 years among Millennium Cohort Study Participants** | | | | | | | |
| --- | --- | --- | --- | --- | --- | --- | --- |
| ***Age 3 years*** | **No. in each exposure group with SDQ ≥17** | **Total SDQ (cut-off ≥17)**  **OR (95% CI)** | **Emotional**  **OR (95% CI)** | **Conduct**  **OR (95% CI)** | **Hyperactivity**  **OR (95% CI)** | **Peer Problems**  **OR (95% CI)** | **Prosocial Behaviour**  **OR (95% CI)** |
| Spontaneous VD | 710 | Ref | Ref | Ref | Ref | Ref | Ref |
| Assisted VD | 107 | 0.76 (0.58, 0.98)^*^ | 0.98 (0.68, 1.41) | 0.83 (0.72, 0.97)^*^ | 0.88 (0.72, 1.08) | 0.99 (0.80, 1.22) | 0.77 (0.57, 1.03) |
| Induced VD | 350 | 1.17 (0.99, 1.38) | 1.08 (0.85, 1.39) | 1.06 (0.95, 1.18) | 1.01 (0.87, 1.17) | 1.02 (0.87, 1.20) | 0.89 (0.71, 1.11) |
| Emergency CS | 83 | 0.74 (0.55, 1.01) | 0.89 (0.60, 1.34) | 0.77 (0.64, 0.92)^*^ | 0.93 (0.73, 1.18) | 0.99 (0.77, 1.26) | 1.08 (0.78, 1.49) |
| Planned CS | 102 | 0.74 (0.56, 0.96)^*^ | 0.93 (0.62, 1.40) | 0.85 (0.72, 1.00) | 0.92 (0.73, 1.14) | 0.78 (0.61, 1.00) | 0.68 (0.48, 0.94)^*^ |
| CS after induction | 99 | 1.14 (0.87, 1.49) | 1.29 (0.87, 1.91) | 0.85 (0.71, 1.02) | 1.00 (0.78, 1.26) | 0.78 (0.59, 1.01) | 0.58 (0.39, 0.86)^*^ |
| ***Age 5 years*** | | | | | | | |
| Spontaneous VD | 372 | Ref | Ref | Ref | Ref | Ref | Ref |
| Assisted VD | 66 | 0.87 (0.63, 1.19) | 1.15 (0.86, 1.55) | 0.63 (0.49, 0.81)^*^ | 0.88 (0.70, 1.11) | 1.04 (0.79, 1.36) | 0.60 (0.33, 1.07) |
| Induced VD | 187 | 1.28 (1.03, 1.59)^*^ | 1.01 (0.80, 1.28) | 0.95 (0.81, 1.12) | 1.05 (0.89, 1.24) | 1.23 (1.01, 1.50)^*^ | 1.14 (0.80, 1.63) |
| Emergency CS | 66 | 1.36 (0.98, 1.89) | 1.02 (0.72, 1.45) | 0.74 (0.55, 1.00) | 1.22 (0.94, 1.57) | 1.46 (1.10, 1.94)^*^ | 1.36 (0.80, 2.31) |
| Planned CS | 44 | 0.65 (0.43, 0.97)^*^ | 0.83 (0.57, 1.20) | 0.56 (0.42, 0.75)^*^ | 0.79 (0.60, 1.04) | 0.88 (0.65, 1.20) | 0.94 (0.55, 1.58) |
| CS after induction | 51 | 1.07 (0.74, 1.54) | 1.12 (0.79, 1.58) | 0.70 (0.52, 0.94)^*^ | 0.77 (0.58, 1.04) | 1.32 (0.97, 1.79) | 0.94 (0.51, 1.71) |
| ***Age 7 years*** | | | | | | | |
| Spontaneous VD | 450 | Ref | Ref | Ref | Ref | Ref | Ref |
| Assisted VD | 82 | 0.76 (0.56, 1.02) | 1.01 (0.76, 1.34) | 0.80 (0.61, 1.04) | 0.79 (0.63, 1.00) | 1.16 (0.90, 1.49) | 0.63 (0.36, 1.11) |
| Induced VD | 226 | 1.24 (1.01, 1.51)^*^ | 1.10 (0.90, 1.35) | 1.18 (0.99, 1.41) | 1.08 (0.92, 1.27) | 1.29 (1.07, 1.55)^*^ | 0.81 (0.53, 1.24) |
| Emergency CS | 65 | 0.85 (0.60, 1.19) | 0.98 (0.71, 1.37) | 0.72 (0.53, 1.00) | 1.17 (0.92, 1.51) | 1.10 (0.81, 1.49) | 0.81 (0.43, 1.53) |
| Planned CS | 66 | 0.83 (0.59, 1.16) | 0.88 (0.63, 1.21) | 0.88 (0.66, 1.16) | 0.82 (0.63, 1.05) | 0.78 (0.57, 1.06) | 0.57 (0.29, 1.11) |
| CS after induction | 55 | 0.84 (0.59, 1.20) | 0.99 (0.72, 1.38) | 0.79 (0.58, 1.07) | 0.83 (0.63, 1.09) | 1.04 (0.77, 1.41) | 1.12 (0.62, 2.04) |
| ***Age 11 years*** | | | | | | | |
| Spontaneous VD | 431 | Ref | Ref | Ref | Ref | Ref | Ref |
| Assisted VD | 76 | 0.84 (0.62, 1.13) | 0.99 (0.78, 1.27) | 0.58 (0.42, 0.79)^*^ | 0.89 (0.69, 1.15) | 1.09 (0.85, 1.40) | 0.88 (0.49, 1.59) |
| Induced VD | 191 | 1.11 (0.89, 1.37) | 1.12 (0.93, 1.35) | 1.06 (0.87, 1.28) | 1.21 (1.01, 1.45)^*^ | 1.14 (0.95, 1.38) | 1.30 (0.86, 1.96) |
| Emergency CS | 73 | 1.16 (0.85, 1.58) | 1.09 (0.83, 1.43) | 1.02 (0.76, 1.38) | 1.36 (1.04, 1.78)^*^ | 1.39 (1.06, 1.81)^*^ | 0.75 (0.34, 1.66) |
| Planned CS | 55 | 0.71 (0.50, 1.02) | 1.14 (0.88, 1.49) | 0.76 (0.56, 1.03) | 0.84 (0.63, 1.13) | 0.85 (0.64, 1.14) | 0.67 (0.31, 1.45) |
| CS after induction | 60 | 1.07 (0.76, 1.50) | 1.56 (1.20, 2.02)^*^ | 0.78 (0.55, 1.09) | 0.86 (0.63, 1.19) | 1.60 (1.23, 2.08)^*^ | 1.15 (0.60, 2.21) |
| ***Age 14 years*** | | | | | | | |
| **Spontaneous VD** | 526 | Ref | Ref | Ref | Ref | Ref | Ref |
| Assisted VD | 90 | 0.78 (0.59, 1.03) | 0.78 (0.60, 1.00) | 0.85 (0.64, 1.12) | 0.67 (0.50, 0.91)^*^ | 1.00 (0.80, 1.24) | 0.97 (0.68, 1.39) |
| Induced VD | 229 | 1.17 (0.96, 1.43) | 1.12 (0.94, 1.34) | 1.10 (0.91, 1.34) | 1.07 (0.87, 1.31) | 1.13 (0.96, 1.34) | 0.74 (0.55, 1.02) |
| Emergency CS | 68 | 0.91 (0.66, 1.26) | 1.06 (0.81, 1.38) | 0.74 (0.53, 1.03) | 1.05 (0.76, 1.44) | 1.13 (0.88, 1.44) | 1.11 (0.72, 1.70) |
| Planned CS | 75 | 0.91 (0.67, 1.24) | 0.91 (0.70, 1.20) | 0.92 (0.69, 1.24) | 0.82 (0.59, 1.13) | 0.83 (0.64, 1.07) | 0.77 (0.48, 1.23) |
| CS after induction | 67 | 0.97 (0.70, 1.33) | 1.31 (1.01, 1.70)^*^ | 0.82 (0.58, 1.14) | 1.02 (0.73, 1.41) | 1.16 (0.90, 1.49) | 1.11 (0.71, 1.72) |
| ***Age 17 years*** | | | | | | | |
| Spontaneous VD | 442 | Ref | Ref | Ref | Ref | Ref | Ref |
| Assisted VD | 81 | 0.86 (0.64, 1.16) | 0.94 (0.74, 1.20) | 0.84 (0.62, 1.14) | 1.24 (0.90, 1.71) | 0.85 (0.67, 1.07) | 1.29 (0.86, 1.95) |
| Induced VD | 196 | 1.26 (1.02, 1.56)^*^ | 1.02 (0.85, 1.23) | 1.23 (0.98, 1.54) | 1.14 (0.88, 1.49) | 0.97 (0.81, 1.15) | 1.08 (0.75, 1.54) |
| Emergency CS | 58 | 0.98 (0.70, 1.39) | 1.03 (0.78, 1.35) | 0.74 (0.50, 1.09) | 0.93 (0.61, 1.43) | 1.11 (0.86, 1.43) | 0.54 (0.28, 1.04) |
| Planned CS | 60 | 1.08 (0.77, 1.52) | 1.20 (0.92, 1.56) | 1.04 (0.74, 1.46) | 0.86 (0.57, 1.31) | 0.95 (0.73, 1.23) | 0.78 (0.43, 1.40) |
| CS after induction | 49 | 0.91 (0.63, 1.31) | 1.11 (0.84, 1.46) | 0.75 (0.50, 1.12) | 0.86 (0.54, 1.36) | 0.83 (0.62, 1.11) | 1.08 (0.63, 1.83) |
| Abbreviations: SDQ, Strengths and Difficulties Questionnaire; OR, odds ratio; 95% CI, 95% confidence interval; VD, vaginal delivery; CS, caesarean section.  *Statistically significant p<0.05. | | | | | | | |

| **Table S2: Association between Mode of Delivery and total Strengths and Difficulties Questionnaire cut-off at ages 3, 5, 7, 11, 14 and 17 years among Millennium Cohort Study Participants by Level of Deprivation** | | | | | | |
| --- | --- | --- | --- | --- | --- | --- |
|  | **Highest deprivation (deciles 1–5)** | | | **Lowest deprivation (deciles 6–10)** | | |
| ***Age 3 years*** | **No. in each exposure group with SDQ ≥17** | **Total SDQ (cut-off ≥17)**  **Crude OR (95% CI)** | **Total SDQ (cut-off ≥17)**  **Adjusted OR^a^ (95% CI)** | **No. in each exposure group with SDQ ≥17** | **Total SDQ (cut-off ≥17)**  **Crude OR (95% CI)** | **Total SDQ (cut-off ≥17)**  **Adjusted OR^a^ (95% CI)** |
| Spontaneous VD | 412 | Ref | Ref | 84 | Ref | Ref |
| Assisted VD | 40 | 0.67 (0.46, 1.00) | 0.79 (0.53, 1.18) | 21 | 1.06 (0.64, 1.75) | 1.13 (0.65, 1.98) |
| Induced VD | 190 | 1.21 (0.98, 1.49) | 1.11 (0.89, 1.38) | 30 | 0.92 (0.59, 1.43) | 0.72 (0.45, 1.15) |
| Emergency CS | 42 | 0.68 (0.45, 1.01) | 0.73 (0.47, 1.12) | 14 | 1.00 (0.54, 1.85) | 0.96 (0.50, 1.83) |
| Planned CS | 47 | 0.81 (0.56, 1.17) | 1.00 (0.68, 1.47) | 11 | 0.67 (0.35, 1.29) | 0.59 (0.31, 1.15) |
| CS after induction | 49 | 1.11 (0.77, 1.60) | 1.27 (0.86, 1.87) | 15 | 1.39 (0.77, 2.50) | 1.51 (0.76, 3.00) |
| ***Age 5 years*** | | | | | | |
| Spontaneous VD | 217 | Ref | Ref | 37 | Ref | Ref |
| Assisted VD | 25 | 0.77 (0.48, 1.23) | 0.93 (0.57, 1.50) | 10 | 1.10 (0.53, 2.25) | 1.26 (0.56, 2.80) |
| Induced VD | 97 | 1.29 (0.98, 1.70) | 1.15 (0.87, 1.53) | 18 | 1.27 (0.70, 2.28) | 1.03 (0.54, 1.94) |
| Emergency CS | 29 | 1.12 (0.71, 1.77) | 1.16 (0.71, 1.90) | 13 | 2.26 (1.17, 4.33)^*^ | 2.18 (1.08, 4.40)^*^ |
| Planned CS | 20 | 0.65 (0.38, 1.13) | 0.77 (0.43, 1.38) | <10 | 0.83 (0.34, 2.02) | 0.72 (0.27, 1.90) |
| CS after induction | 25 | 1.01 (0.62, 1.64) | 1.04 (0.61, 1.74) | <10 | 1.33 (0.55, 3.20) | 1.36 (0.52, 3.54) |
| ***Age 7 years*** | | | | | | |
| Spontaneous VD | 240 | Ref | Ref | 68 | Ref | Ref |
| Assisted VD | 28 | 0.76 (0.48, 1.19) | 0.83 (0.51, 1.34) | <10 | 0.58 (0.28, 1.19) | 0.56 (0.25, 1.24) |
| Induced VD | 119 | 1.40 (1.08, 1.81)^*^ | 1.33 (1.01, 1.74)^*^ | 22 | 0.94 (0.57, 1.55) | 0.88 (0.52, 1.49) |
| Emergency CS | 26 | 0.66 (0.40, 1.09) | 0.70 (0.41, 1.17) | 12 | 1.08 (0.55, 2.11) | 1.00 (0.48, 2.09) |
| Planned CS | 26 | 0.89 (0.54, 1.45) | 1.12 (0.68, 1.84) | <10 | 0.78 (0.38, 1.60) | 0.86 (0.40, 1.81) |
| CS after induction | 27 | 0.84 (0.52, 1.35) | 0.86 (0.51, 1.43) | <10 | 0.85 (0.38, 1.91) | 0.94 (0.39, 2.29) |
| ***Age 11 years*** | | | | | | |
| Spontaneous VD | 227 | Ref | Ref | 69 | Ref | Ref |
| Assisted VD | 22 | 0.63 (0.39, 1.02) | 0.68 (0.41, 1.14) | 18 | 1.11 (0.64, 1.91) | 1.24 (0.68, 2.24) |
| Induced VD | 86 | 0.99 (0.75, 1.32) | 0.90 (0.67, 1.20) | 27 | 1.17 (0.73, 1.87) | 1.04 (0.65, 1.68) |
| Emergency CS | 37 | 1.17 (0.77, 1.77) | 1.22 (0.79, 1.89) | 15 | 1.34 (0.73, 2.47) | 1.29 (0.67, 2.51) |
| Planned CS | 24 | 0.67 (0.40, 1.12) | 0.75 (0.45, 1.25) | 11 | 0.90 (0.46, 1.75) | 0.85 (0.43, 1.69) |
| CS after induction | 24 | 1.09 (0.68, 1.75) | 1.07 (0.65, 1.77) | <10 | 1.05 (0.51, 2.16) | 1.30 (0.58, 2.91) |
| ***Age 14 years*** | | | | | | |
| Spontaneous VD | 293 | Ref | Ref | 80 | Ref | Ref |
| Assisted VD | 28 | 0.60 (0.38, 0.94)^*^ | 0.73 (0.46, 1.16) | 18 | 0.95 (0.55, 1.65) | 1.11 (0.62, 1.98) |
| Induced VD | 113 | 1.07 (0.83, 1.39) | 0.99 (0.76, 1.29) | 36 | 1.46 (0.96, 2.21) | 1.41 (0.91, 2.18) |
| Emergency CS | 29 | 0.78 (0.49, 1.24) | 0.86 (0.53, 1.40) | 16 | 1.25 (0.70, 2.23) | 1.23 (0.65, 2.31) |
| Planned CS | 35 | 0.85 (0.55, 1.32) | 0.99 (0.63, 1.56) | 14 | 1.15 (0.63, 2.09) | 1.14 (0.60, 2.16) |
| CS after induction | 29 | 0.89 (0.56, 1.39) | 0.89 (0.55, 1.44) | <10 | 0.95 (0.46, 1.96) | 1.03 (0.50, 2.14) |
| ***Age 17 years*** | | | | | | |
| Spontaneous VD | 243 | Ref | Ref | 61 | Ref | Ref |
| Assisted VD | 34 | 0.87 (0.57, 1.33) | 1.07 (0.68, 1.67) | 12 | 0.91 (0.48, 1.74) | 1.17 (0.60, 2.28) |
| Induced VD | 94 | 1.19 (0.89, 1.60) | 1.17 (0.87, 1.57) | 32 | 1.56 (0.99, 2.47) | 1.45 (0.90, 2.32) |
| Emergency CS | 34 | 1.07 (0.68, 1.67) | 1.20 (0.74, 1.95) | 12 | 1.21 (0.62, 2.37) | 1.18 (0.58, 2.37) |
| Planned CS | 28 | 1.11 (0.68, 1.79) | 1.29 (0.79, 2.10) | 15 | 1.60 (0.87, 2.93) | 1.75 (0.92, 3.33) |
| CS after induction | 27 | 0.97 (0.59, 1.57) | 1.04 (0.64, 1.71) | <10 | 0.90 (0.39, 2.05) | 1.20 (0.51, 2.80) |
| Abbreviations: SDQ, Strengths and Difficulties Questionnaire; OR, odds ratio; 95% CI, 95% confidence interval; VD, vaginal delivery; CS, caesarean section.  ^a^Adjusted for maternal age, maternal education, maternal smoking status, maternal alcohol consumption during pregnancy, pre-pregnancy body mass index, household income, small for gestational age, infant sex, parity, hypertensive disorders of pregnancy and maternal depression/serious anxiety.  *Statistically significant p<0.05. | | | | | | |

| **Table S3: Association between Mode of Delivery and total Strengths and Difficulties Questionnaire cut-off at ages 3, 5, 7, 11, 14 and 17 years among Millennium Cohort Study Participants (excluding preterm births)** | | | |
| --- | --- | --- | --- |
| ***Age 3 years*** | **No. in each exposure group with SDQ ≥17** | **Total SDQ (cut-off ≥17)**  **Crude OR (95% CI)** | **Total SDQ (cut-off ≥17)**  **Adjusted OR^a^ (95% CI)** |
| Spontaneous VD | 652 | Ref | Ref |
| Assisted VD | 105 | 0.82 (0.63, 1.07) | 0.97 (0.73, 1.29) |
| Induced VD | 322 | 1.16 (0.97, 1.37) | 1.01 (0.84, 1.20) |
| Emergency CS | 52 | 0.64 (0.44, 0.93)^*^ | 0.71 (0.48, 1.06) |
| Planned CS | 94 | 0.76 (0.58, 1.01) | 0.94 (0.70, 1.25) |
| CS after induction | 81 | 1.01 (0.75, 1.37) | 1.14 (0.82, 1.58) |
| ***Age 5 years*** | | | |
| Spontaneous VD | 342 | Ref | Ref |
| Assisted VD | 61 | 0.88 (0.63, 1.23) | 1.04 (0.73, 1.49) |
| Induced VD | 181 | 1.34 (1.07, 1.68)^*^ | 1.14 (0.91, 1.43) |
| Emergency CS | 43 | 1.17 (0.78, 1.75) | 1.40 (0.92, 2.12) |
| Planned CS | 41 | 0.71 (0.47, 1.07) | 0.78 (0.50, 1.20) |
| CS after induction | 41 | 0.93 (0.62, 1.42) | 0.94 (0.61, 1.46) |
| ***Age 7 years*** | | | |
| Spontaneous VD | 412 | Ref | Ref |
| Assisted VD | 72 | 0.71 (0.52, 0.98)^*^ | 0.75 (0.53, 1.07) |
| Induced VD | 208 | 1.27 (1.03, 1.56)^*^ | 1.15 (0.92, 1.42) |
| Emergency CS | 46 | 0.76 (0.51, 1.14) | 0.81 (0.53, 1.25) |
| Planned CS | 62 | 0.90 (0.64, 1.27) | 1.10 (0.77, 1.56) |
| CS after induction | 48 | 0.80 (0.55, 1.17) | 0.84 (0.56, 1.25) |
| ***Age 11 years*** | | | |
| Spontaneous VD | 392 | Ref | Ref |
| Assisted VD | 73 | 0.87 (0.64, 1.19) | 0.98 (0.70, 1.36) |
| Induced VD | 181 | 1.14 (0.92, 1.42) | 1.02 (0.82, 1.28) |
| Emergency CS | 46 | 0.94 (0.64, 1.39) | 1.00 (0.67, 1.51) |
| Planned CS | 52 | 0.71 (0.49, 1.03) | 0.78 (0.54, 1.13) |
| CS after induction | 52 | 0.98 (0.68, 1.43) | 1.01 (0.68, 1.51) |
| ***Age 14 years*** | | | |
| Spontaneous VD | 482 | Ref | Ref |
| Assisted VD | 86 | 0.83 (0.62, 1.11) | 0.99 (0.73, 1.33) |
| Induced VD | 215 | 1.21 (0.98, 1.48) | 1.10 (0.89, 1.36) |
| Emergency CS | 47 | 0.77 (0.52, 1.15) | 0.88 (0.58, 1.32) |
| Planned CS | 69 | 0.95 (0.69, 1.31) | 1.08 (0.77, 1.50) |
| CS after induction | 52 | 0.82 (0.57, 1.19) | 0.87 (0.59, 1.27) |
| ***Age 17 years*** | | | |
| Spontaneous VD | 400 | Ref | Ref |
| Assisted VD | 74 | 0.85 (0.62, 1.16) | 1.01 (0.73, 1.40) |
| Induced VD | 185 | 1.29 (1.04, 1.62)^*^ | 1.22 (0.97, 1.53) |
| Emergency CS | 45 | 1.00 (0.68, 1.49) | 1.15 (0.76, 1.75) |
| Planned CS | 53 | 1.09 (0.76, 1.55) | 1.25 (0.87, 1.79) |
| CS after induction | 38 | 0.84 (0.56, 1.27) | 0.93 (0.62, 1.41) |
| Abbreviations: SDQ, Strengths and Difficulties Questionnaire; OR, odds ratio; 95% CI, 95% confidence interval; VD, vaginal delivery; CS, caesarean section.  ^a^Adjusted for maternal age, maternal education, maternal smoking status, maternal alcohol consumption during pregnancy, pre-pregnancy body mass index, household income, small for gestational age, infant sex, parity, hypertensive disorders of pregnancy and maternal depression/serious anxiety.  *Statistically significant p<0.05. | | | |

| **Table S4: Association between Mode of Delivery and total Strengths and Difficulties Questionnaire cut-off at ages 3, 5, 7, 11, 14 and 17 years among Millennium Cohort Study Participants by Presence or Absence of Maternal Depression/Serious Anxiety** | | | | | | |
| --- | --- | --- | --- | --- | --- | --- |
|  | **No maternal depression/serious anxiety** | | | **Maternal depression/serious anxiety** | | |
| ***Age 3 years*** | **No. in each exposure group with SDQ ≥17** | **Total SDQ (cut-off ≥17)**  **Crude OR (95% CI)** | **Total SDQ (cut-off ≥17)**  **Adjusted OR^a^ (95% CI)** | **No. in each exposure group with SDQ ≥17** | **Total SDQ (cut-off ≥17)**  **Crude OR (95% CI)** | **Total SDQ (cut-off ≥17)**  **Adjusted OR^a^ (95% CI)** |
| Spontaneous VD | 486 | Ref | Ref | 224 | Ref | Ref |
| Assisted VD | 70 | 0.70 (0.51, 0.97)^*^ | 0.87 (0.62, 1.23) | 37 | 0.91 (0.58, 1.43) | 0.97 (0.59, 1.60) |
| Induced VD | 214 | 1.04 (0.84, 1.27) | 0.91 (0.74, 1.13) | 135 | 1.32 (1.00, 1.74) | 1.22 (0.90, 1.64) |
| Emergency CS | 51 | 0.69 (0.47, 1.00) | 0.74 (0.50, 1.10) | 32 | 0.88 (0.52, 1.49) | 0.93 (0.54, 1.59) |
| Planned CS | 57 | 0.57 (0.40, 0.81)^*^ | 0.71 (0.49, 1.02) | 45 | 1.04 (0.68, 1.59) | 1.27 (0.81, 1.97) |
| CS after induction | 67 | 1.23 (0.89, 1.70) | 1.45 (1.02, 2.06)^*^ | 32 | 0.92 (0.56, 1.53) | 1.01 (0.58, 1.76) |
| ***Age 5 years*** | | | | | | |
| Spontaneous VD | 249 | Ref | Ref | 123 | Ref | Ref |
| Assisted VD | 36 | 0.75 (0.49, 1.13) | 1.02 (0.65, 1.59) | 30 | 1.14 (0.68, 1.91) | 1.19 (0.68, 2.08) |
| Induced VD | 110 | 1.22 (0.93, 1.61) | 1.08 (0.82, 1.44) | 77 | 1.28 (0.90, 1.82) | 1.17 (0.81, 1.70) |
| Emergency CS | 33 | 1.04 (0.66, 1.62) | 1.24 (0.78, 1.98) | 33 | 2.03 (1.23, 3.37)^*^ | 2.04 (1.18, 3.50)^*^ |
| Planned CS | 25 | 0.52 (0.30, 0.89)^*^ | 0.62 (0.36, 1.08) | 19 | 0.81 (0.44, 1.49) | 0.93 (0.48, 1.81) |
| CS after induction | 30 | 1.10 (0.70, 1.74) | 1.23 (0.75, 2.00) | 21 | 0.98 (0.52, 1.84) | 0.91 (0.47, 1.72) |
| ***Age 7 years*** | | | | | | |
| Spontaneous VD | 291 | Ref | Ref | 159 | Ref | Ref |
| Assisted VD | 51 | 0.78 (0.54, 1.14) | 0.93 (0.62, 1.39) | 31 | 0.74 (0.45, 1.22) | 0.61 (0.35, 1.07) |
| Induced VD | 138 | 1.21 (0.94, 1.56) | 1.15 (0.89, 1.49) | 87 | 1.17 (0.84, 1.64) | 1.12 (0.78, 1.60) |
| Emergency CS | 37 | 0.73 (0.47, 1.15) | 0.83 (0.51, 1.34) | 28 | 1.05 (0.61, 1.82) | 1.03 (0.59, 1.81) |
| Planned CS | 36 | 0.68 (0.43, 1.07) | 0.89 (0.56, 1.43) | 30 | 1.06 (0.63, 1.77) | 1.21 (0.71, 2.07) |
| CS after induction | 30 | 0.76 (0.48, 1.20) | 0.84 (0.52, 1.38) | 25 | 0.99 (0.56, 1.75) | 0.90 (0.50, 1.64) |
| ***Age 11 years*** | | | | | | |
| Spontaneous VD | 267 | Ref | Ref | 164 | Ref | Ref |
| Assisted VD | 52 | 0.98 (0.68, 1.40) | 1.10 (0.75, 1.60) | 24 | 0.63 (0.36, 1.10) | 0.65 (0.35, 1.19) |
| Induced VD | 109 | 1.01 (0.77, 1.33) | 0.92 (0.70, 1.22) | 81 | 1.16 (0.83, 1.63) | 1.07 (0.75, 1.51) |
| Emergency CS | 43 | 1.16 (0.78, 1.72) | 1.20 (0.80, 1.82) | 30 | 1.18 (0.71, 1.97) | 1.15 (0.67, 1.97) |
| Planned CS | 28 | 0.57 (0.35, 0.94)^*^ | 0.67 (0.40, 1.10) | 27 | 0.93 (0.55, 1.59) | 0.91 (0.53, 1.57) |
| CS after induction | 37 | 1.10 (0.73, 1.67) | 1.16 (0.75, 1.81) | 23 | 1.01 (0.56, 1.83) | 1.01 (0.53, 1.91) |
| ***Age 14 years*** | | | | | | |
| Spontaneous VD | 348 | Ref | Ref | 178 | Ref | Ref |
| Assisted VD | 56 | 0.78 (0.55, 1.11) | 1.10 (0.75, 1.60) | 34 | 0.81 (0.50, 1.31) | 0.87 (0.53, 1.43) |
| Induced VD | 136 | 1.09 (0.85, 1.40) | 0.92 (0.70, 1.22) | 93 | 1.24 (0.89, 1.72) | 1.17 (0.82, 1.65) |
| Emergency CS | 46 | 1.02 (0.69, 1.50) | 1.20 (0.80, 1.82) | 22 | 0.72 (0.40, 1.28) | 0.74 (0.40, 1.35) |
| Planned CS | 44 | 0.94 (0.63, 1.38) | 0.67 (0.40, 1.10) | 31 | 0.84 (0.51, 1.39) | 0.94 (0.56, 1.59) |
| CS after induction | 43 | 1.05 (0.71, 1.55) | 1.16 (0.75, 1.81) | 24 | 0.79 (0.44, 1.42) | 0.76 (0.43, 1.35) |
| ***Age 17 years*** | | | | | | |
| Spontaneous VD | 307 | Ref | Ref | 135 | Ref | Ref |
| Assisted VD | 52 | 0.80 (0.56, 1.16) | 1.05 (0.72, 1.53) | 29 | 1.08 (0.64, 1.82) | 1.22 (0.71, 2.11) |
| Induced VD | 110 | 1.02 (0.77, 1.34) | 0.97 (0.73, 1.28) | 86 | 1.74 (1.22, 2.49)^*^ | 1.77 (1.22, 2.57)^*^ |
| Emergency CS | 40 | 0.99 (0.65, 1.50) | 1.11 (0.72, 1.71) | 18 | 0.98 (0.52, 1.84) | 0.99 (0.50, 1.97) |
| Planned CS | 38 | 0.89 (0.58, 1.37) | 1.01 (0.65, 1.55) | 22 | 1.50 (0.86, 2.60) | 2.06 (1.15, 3.68)^*^ |
| CS after induction | 30 | 0.74 (0.47, 1.18) | 0.88 (0.55, 1.40) | 19 | 1.32 (0.71, 2.45) | 1.40 (0.75, 2.63) |
| Abbreviations: SDQ, Strengths and Difficulties Questionnaire; OR, odds ratio; 95% CI, 95% confidence interval; VD, vaginal delivery; CS, caesarean section.  ^a^Adjusted for maternal age, maternal education, maternal smoking status, maternal alcohol consumption during pregnancy, pre-pregnancy body mass index, household income, small for gestational age, infant sex, parity and hypertensive disorders of pregnancy.  *Statistically significant p<0.05. | | | | | | |

| **Table S5: Association between Mode of Delivery and total Strengths and Difficulties Questionnaire cut-off at ages 3, 5, 7, 11, 14 and 17 years among Millennium Cohort Study Participants (among participants selected based on having complete exposure, covariate and outcome data)** | | |
| --- | --- | --- |
| ***Age 3 years*** | **No. in each exposure group with SDQ ≥17** | **Total SDQ (cut-off ≥17)**  **Adjusted OR^a^ (95% CI)** |
| Spontaneous VD | 169 | Ref |
| Assisted VD | 34 | 1.13 (0.69, 1.82) |
| Induced VD | 77 | 1.10 (0.78, 1.55) |
| Emergency CS | 18 | 0.77 (0.42, 1.41) |
| Planned CS | 23 | 0.71 (0.41, 1.23) |
| CS after induction | 23 | 1.30 (0.73, 2.29) |
| ***Age 5 years*** | | |
| Spontaneous VD | 92 | Ref |
| Assisted VD | 19 | 0.93 (0.49, 1.77) |
| Induced VD | 42 | 1.03 (0.65, 1.65) |
| Emergency CS | 16 | 1.40 (0.73, 2.67) |
| Planned CS | 14 | 1.11 (0.51, 2.42) |
| CS after induction | <10 | 0.50 (0.20, 1.21) |
| ***Age 7 years*** | | |
| Spontaneous VD | 115 | Ref |
| Assisted VD | 24 | 0.91 (0.50, 1.64) |
| Induced VD | 51 | 1.15 (0.75, 1.75) |
| Emergency CS | 20 | 1.06 (0.56, 2.01) |
| Planned CS | 18 | 1.12 (0.58, 2.18) |
| CS after induction | 19 | 1.52 (0.80, 2.86) |
| ***Age 11 years*** | | |
| Spontaneous VD | 165 | Ref |
| Assisted VD | 27 | 0.86 (0.51, 1.45) |
| Induced VD | 59 | 0.79 (0.55, 1.14) |
| Emergency CS | 27 | 1.10 (0.65, 1.84) |
| Planned CS | 19 | 0.87 (0.48, 1.57) |
| CS after induction | 21 | 1.20 (0.68, 2.13) |
| ***Age 14 years*** | | |
| Spontaneous VD | 175 | Ref |
| Assisted VD | 39 | 1.15 (0.74, 1.80) |
| Induced VD | 70 | 0.95 (0.67, 1.34) |
| Emergency CS | 29 | 0.96 (0.57, 1.63) |
| Planned CS | 28 | 1.12 (0.67, 1.87) |
| CS after induction | 27 | 1.51 (0.92, 2.50) |
| ***Age 17 years*** | | |
| Spontaneous VD | 183 | Ref |
| Assisted VD | 36 | 1.18 (0.76, 1.84) |
| Induced VD | 85 | 1.16 (0.84, 1.60) |
| Emergency CS | 29 | 1.30 (0.79, 2.14) |
| Planned CS | 30 | 1.44 (0.90, 2.32) |
| CS after induction | 28 | 1.48 (0.92, 2.39) |
| Abbreviations: SDQ, Strengths and Difficulties Questionnaire; OR, odds ratio; 95% CI, 95% confidence interval; VD, vaginal delivery; CS, caesarean section.  ^a^Adjusted for maternal age, maternal education, maternal smoking status, maternal alcohol consumption during pregnancy, pre-pregnancy body mass index, household income, small for gestational age, infant sex, parity, hypertensive disorders of pregnancy and maternal depression/serious anxiety. | | |

| **Table S6: Association between mother and child characteristics and loss to follow-up at age 17 years among Millennium Cohort Study Participants** | |
| --- | --- |
|  | **Loss to follow-up at age 17 years**  **OR (95% CI)** |
| *Maternal age* | 0.95 (0.94, 0.96) |
| *Maternal education* |  |
| Diploma or above | ref |
| Less than O level | 2.91 (2.60, 3.25)^*^ |
| O level | 1.84 (1.69, 2.02)^*^ |
| A level | 1.37 (1.20, 1.57)^*^ |
| *Maternal smoking status* |  |
| Non-smoker | ref |
| Quit during pregnancy | 1.33 (1.19, 1.47)^*^ |
| Smoked during pregnancy | 1.74 (1.60, 1.90)^*^ |
| *Maternal alcohol consumption during pregnancy* |  |
| Yes | 0.77 (0.71, 0.83)^*^ |
| *Pre-pregnancy BMI* |  |
| Normal weight | ref |
| Underweight | 1.45 (1.24, 1.70)^*^ |
| Overweight | 0.99 (0.90, 1.09) |
| Obese | 0.96 (0.84, 1.10) |
| *Household income* |  |
| Highest quintile | ref |
| Fourth quintile | 1.15 (1.02, 1.29)^*^ |
| Third quintile | 1.49 (1.33, 1.67)^*^ |
| Second quintile | 1.85 (1.65, 2.07)^*^ |
| Lowest quintile | 2.81 (2.51, 3.15)^*^ |
| *SGA* |  |
| Yes | 1.15 (1.02, 1.30)^*^ |
| *Infant sex* |  |
| Male | 1.12 (1.05, 1.20)^*^ |
| *Parity* |  |
| First born | 1.06 (0.99, 1.14) |
| *Hypertensive disorders of pregnancy* |  |
| Yes | 0.95 (0.83, 1.09) |
| *Maternal depression/serious anxiety* |  |
| Yes | 1.13 (1.04, 1.23)^*^ |
| *Total SDQ cut-off at age 3 years* |  |
| Total SDQ (cut-off <17) | ref |
| Total SDQ (cut-off ≥17) | 1.59 (1.39, 1.81)^*^ |
| Abbreviations: SDQ, Strengths and Difficulties Questionnaire; OR, odds ratio; 95% CI, 95% confidence interval; BMI, body mass index; SGA, small for gestational age  *Statistically significant p<0.05. | |

| **Table S7: Association between Mode of Delivery and total Strengths and Difficulties Questionnaire cut-off at ages 3, 5, 7, 11, 14 and 17 years among Millennium Cohort Study Participants (including attrition weight for age 17)** | |
| --- | --- |
| ***Age 3 years*** | **Total SDQ (cut-off ≥17)**  **Adjusted OR^a^ (95% CI)** |
| Spontaneous VD | Ref |
| Assisted VD | 0.87 (0.56, 1.35) |
| Induced VD | 0.88 (0.65, 1.19) |
| Emergency CS | 0.73 (0.46, 1.16) |
| Planned CS | 0.72 (0.49, 1.05) |
| CS after induction | 1.57 (0.90, 2.73) |
| ***Age 5 years*** | |
| Spontaneous VD | Ref |
| Assisted VD | 0.91 (0.53, 1.55) |
| Induced VD | 0.87 (0.63, 1.22) |
| Emergency CS | 1.57 (0.98, 2.53) |
| Planned CS | 0.66 (0.35, 1.26) |
| CS after induction | 0.85 (0.40, 1.80) |
| ***Age 7 years*** | |
| Spontaneous VD | Ref |
| Assisted VD | 0.93 (0.61, 1.41) |
| Induced VD | 1.07 (0.78, 1.46) |
| Emergency CS | 0.65 (0.43, 1.00) |
| Planned CS | 0.96 (0.61, 1.50) |
| CS after induction | 0.81 (0.50, 1.30) |
| ***Age 11 years*** | |
| Spontaneous VD | Ref |
| Assisted VD | 0.91 (0.59, 1.40) |
| Induced VD | 1.33 (0.94, 1.88) |
| Emergency CS | 1.53 (0.85, 2.75) |
| Planned CS | 0.67 (0.42, 1.04) |
| CS after induction | 1.34 (0.79, 2.27) |
| ***Age 14 years*** | |
| Spontaneous VD | Ref |
| Assisted VD | 0.89 (0.60, 1.31) |
| Induced VD | 1.08 (0.80, 1.46) |
| Emergency CS | 1.40 (0.79, 2.47) |
| Planned CS | 1.09 (0.76, 1.57) |
| CS after induction | 0.83 (0.57, 1.19) |
| ***Age 17 years*** | |
| Spontaneous VD | Ref |
| Assisted VD | 1.14 (0.74, 1.73) |
| Induced VD | 1.28 (0.84, 1.94) |
| Emergency CS | 0.86 (0.57, 1.28) |
| Planned CS | 1.26 (0.81, 1.95) |
| CS after induction | 0.88 (0.57, 1.35) |
| Abbreviations: SDQ, Strengths and Difficulties Questionnaire; OR, odds ratio; 95% CI, 95% confidence interval; VD, vaginal delivery; CS, caesarean section.  ^a^Adjusted for maternal age, maternal education, maternal smoking status, maternal alcohol consumption during pregnancy, pre-pregnancy body mass index, household income, small for gestational age, infant sex, parity, hypertensive disorders of pregnancy and maternal depression/serious anxiety. | |

| **Table S8: Association between any Caesarean Section and total Strengths and Difficulties Questionnaire cut-off at ages 3, 5, 7, 11, 14 and 17 years among Millennium Cohort Study Participants** | | |
| --- | --- | --- |
| ***Age 3 years*** | **No. in each exposure group with SDQ ≥17** | **Total SDQ (cut-off ≥17)**  **Adjusted OR^a^ (95% CI)** |
| Any VD | 1167 | Ref |
| Any CS | 284 | 0.97 (0.81, 1.16) |
| ***Age 5 years*** | | |
| Any VD | 625 | Ref |
| Any CS | 161 | 1.04 (0.82, 1.31) |
| ***Age 7 years*** | | |
| Any VD | 758 | Ref |
| Any CS | 186 | 0.91 (0.74, 1.14) |
| ***Age 11 years*** | | |
| Any VD | 698 | Ref |
| Any CS | 188 | 1.02 (0.83, 1.26) |
| ***Age 14 years*** | | |
| Any VD | 845 | Ref |
| Any CS | 210 | 1.00 (0.82, 1.23) |
| ***Age 17 years*** | | |
| Any VD | 719 | Ref |
| Any CS | 167 | 1.07 (0.86, 1.33) |
| Abbreviations: SDQ, Strengths and Difficulties Questionnaire; OR, odds ratio; 95% CI, 95% confidence interval; VD, vaginal delivery; CS, caesarean section.  ^a^Adjusted for maternal age, maternal education, maternal smoking status, maternal alcohol consumption during pregnancy, pre-pregnancy body mass index, household income, small for gestational age, infant sex, parity, hypertensive disorders of pregnancy and maternal depression/serious anxiety. | | |
